# The effect of luteinizing hormone changes in GnRH antagonist protocol on the outcome of controlled ovarian hyperstimulation and embryo transfer

**DOI:** 10.1186/s12884-023-05916-8

**Published:** 2023-08-23

**Authors:** Jing-Shuang Zhou, Jian-Hong Chen, Fei-Fei Tang, Jian-Ping Ou, Xin Tao, Liu-Hong Cai

**Affiliations:** Center for Reproductive Medicine, The Third Affiliated Hospital, Sun Yat-sen University, Guangzhou, Guangdong Province China

**Keywords:** Gonadotropin-releasing hormone antagonist protocol, Controlled ovarian hyperstimulation, Fresh embryo transfer, LH level

## Abstract

**Backgroud:**

To investigate the effect of Luteinizing hormone (LH) level changes on the outcomes of controlled ovarian hyperstimulation (COH) and embryo transfer (ET) in gonadotropin-releasing hormone antagonist (GnRH-ant) protocol.

**Methods:**

A total of 721 patients undergoing GnRH-ant protocol COH for the first IVF/ICSI cycles were retrospectively analyzed. COH process were divided into 2 stages, before (stage 1) and after (stage 2) the GnRH-ant initiation, and each with 5 groups basing on LH levels: LH decreased more than 50% (groups A1, A2), decreased 25-50% (groups B1, B2), change less than 25% (groups C1, C2), increased 25-50% (groups D1, D2), and increased more than 50% (groups E1, E2).

**Results:**

There were no significant differences among groups of stage1 regarding COH and ET outcomes. For stage 2, the more obvious the decrease of LH level, the more the number of oocytes retrieved, mature oocytes, fertilized oocytes, embryos cleavaged and the numbers of embryo available (P < 0.05), but without significant differences regarding ET outcomes. We also found the freeze-all rate in Group A2 was higher (P < 0.001).

**Conclusion:**

LH level changes before GnRH-ant addition were not related to COH and ET outcomes. LH level changes after the addition of GnRH-ant were related to the outcome of COH, and no significant differences were found relating to ET outcomes.

**Supplementary Information:**

The online version contains supplementary material available at 10.1186/s12884-023-05916-8.

## Backgroud

Luteinizing hormone (LH) is a glycoprotein hormone secreted by the gonadotropin cells of the anterior pituitary gland, which plays an important role in hormone generation, ovulation promotion and luteinization [1]. Gonadotropin-releasing hormone antagonist (GnRH-ant) protocol has been widely used in assisted reproduction in recent years due to its advantages of non-flare up effect, rapidly and effectively inhibiting the LH surge, reduction of ovarian hyperstimulation syndrome (OHSS) incidence and short treatment period [2]. LH level changes diversitily during COH, most LH levels spontaneously decrease before the administration of antagonist, while about 1/3 LH levels increased, and there are differences in LH level changes between before and after the administration of antagonist [2].

Excessive or insufficient LH levels as well as significant rise or decrease of LH levels will reduce the clinical pregnancy rate [3–5]. While the LH level decreased achieved better COH outcomes than increased ones [6]. Meanwhile, there was no difference in clinical outcomes with different LH levels or LH level changes in similar articles [7–9].

Up to date, there is no unified conclusion on the effect of LH level changes on COH and fresh ET outcomes in GnRH-ant protocol. Most of published studies have focused on the significant changes of LH level at a single time point, here we study the LH level changes before and after the initiation of antagonist during COH. The purpose of this study was to explore the effect of LH level changes on COH and ET outcomes.

## Materials and methods

### Subjects

A retrospective study was performed, analyzing data from 721 the first in vitro fertilization (IVF) or intracytoplasmic sperm injection (ICSI) cycles performed at Center for Reproductive Medicine, The Third Affiliated Hospital, Sun Yat-sen University (China, Guangdong) during the year 2019.

**Fig. 1:**
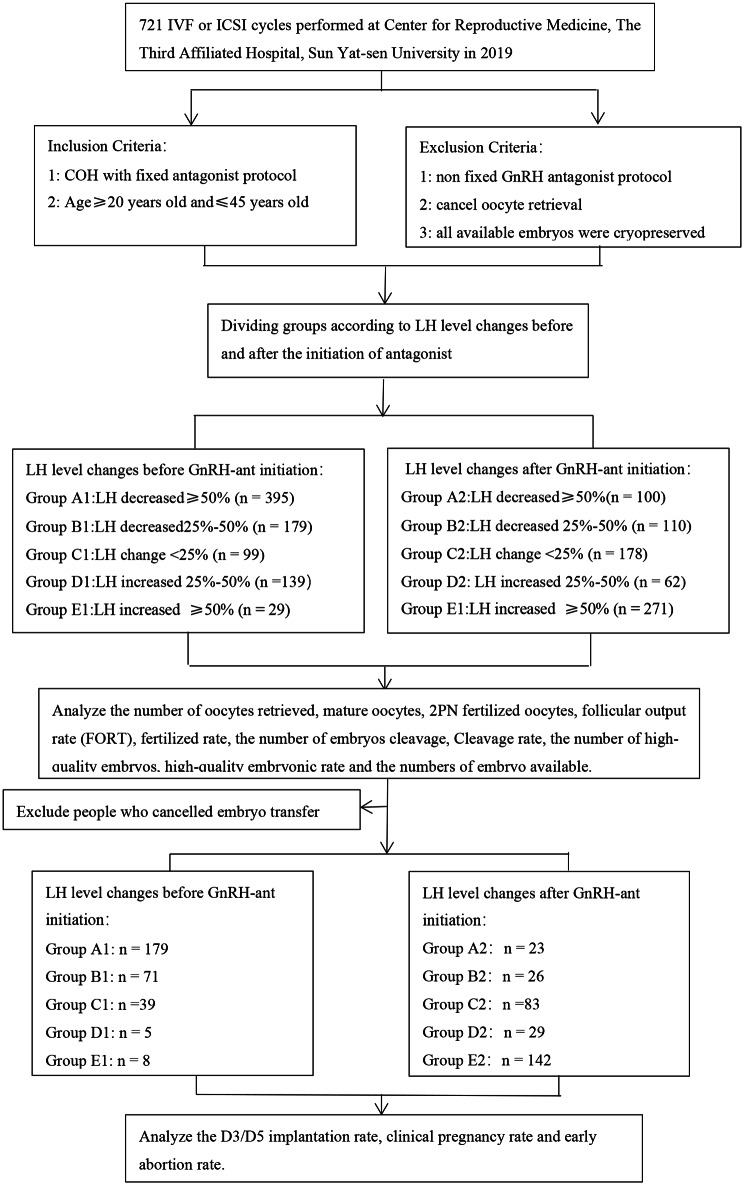
Schematic diagram of research route

The research steps are shown in the Fig. 1.

The study was approved by the Ethics Committee Review Board of The Third Affiliated Hospital, Sun Yat-sen University (No: [2021]02-246-01).

### Inclusion criteria

Patients who underwent ovarian stimulation with GnRH-ant (ganirelix [Ganirest®, 0.25 mg; MSD] or cetrorelix [Cetrotide®, 0.25 mg; Merck]) protocol to suppress the pituitary and patients aged 20–45 years old were enrolled in this study.

### Exclusion criteria

Patients who underwent ovarian stimulation with non-GnRH antagonist protocol, with history of hydrosalpinx, uterine malformation and uterine adhesions, endometriosis, endometrial polyps, polycystic ovary syndrome and other diseases or a concurrent medical condition that have to cancel oocyte retrieval were excluded in this study.

### Protocol

GnRH-ant protocol was applied in this study. COH began on day 2 or 3 of a menstrual period with initial injections of 150–300IU of gonadotropin (Gn) (Gonal F, 450IU; Merck/ Follistim, 300IU; MSD). Antagonist ganirelix (Ganirest, 0.25 mg; MSD) or cetrorelix (Cetrotide, 0.25 mg; Merck) was administered 4 days later according to fixed GnRH antagonist protocol. Drugs are injected at a fixed time every morning. Recombinant human chorionic gonadotropin (rhCG) (Ovitrelle, 250 ug; Merck) or human chorionic gonadotropin (hCG, 10000IU; Lizhu Pharmaceuticals) was administered once when three leading follicles reached ≥ 17 mm mean diameter or one leading follicle reached ≥ 18 mm mean diameter were observed, oocyte retrieval was performed 34–36 h later. Blood samples were taken to measure hormone levels (Follicle stimulating hormone (FSH), Luteinizing hormone (LH), Estrogen (E2), Progesterone (P)) on day 1 of ovarian stimulation (day 2–3 of menstrual cycle), day 1 of the antagonist injection (day 6–7 of menstrual cycle) and trigger day, meanwhile B-ultrasounds were performed for follicular size and number. After oocyte retrieval, IVF or ICSI was routinely performed and embryo culture was performed after fertilization. According to Veeck’s criteria and Gardner’s scoring criteria, normal fertilized embryos with a cell count of 7, 8 and 9 on the third day after fertilization, and blastocysts with a stage 3 or more in their inner cell mass and trophoblast cell scores ≥ B on the fifth day, or blastocysts with a stage 4 or more in their inner cell mass and trophoblast cell scores ≥ B on the sixth day, were defined as high-quality embryos.

COH process were divided into 2 stages, before (stage 1) and after (stage 2) the GnRH-ant initiation, and each with 5 groups basing on LH levels: LH decreased more than 50% (A1, A2), decreased 25-50% (B1, B2), change less than 25% (C1, C2), increased 25-50% (D1, D2), and increased more than 50% (E1, E2).

Calculation method: LH changes before GnRH-ant initiation: (LH level on the fifth day of ovulation stimulation - LH level on the first day of ovulation stimulation)/ LH level on the first day of ovulation stimulation. LH changes after GnRH-ant initiation: (LH level on trigger day - LH level on the fifth day of ovulation stimulation)/ LH level on the fif day of ovulation stimulation.

### Statistical analysis

Continuous variables were presented as means ± standard deviation (SD), categorical variables were presented as percentage. For quantitative data, Kruskal - Wallis tests were used for statistical analysis, while for qualitative data, Fisher’s Exact test or Chi-Square test was used for statistical analysis. For multivariate studies, multiple linear regression models were used for statistical analysis. The significance of all statistical results was set at P < 0.05, and statistical analysis was performed using SPSS software (SPSS Version 25.0.0.0, IBM Corp., USA).

## Results

### The effect of LH level changes before the initiation of GnRH-ant on the outcome of COH and ET

There was a total of 721 first IVF/ICSI patient-cycles with 395 cycles (54.8%) in LH decreased more than 50% before the initiation of GnRH-ant (A1), 179 cycles (24.8%) in LH decreased 25-50% (B1), 99 cycles (13.7%) in LH change less than 25%(C1), 19 cycles (2.6%) in LH increased 25-50% (D1) and 29 cycles (4.0%) in LH increased more than 50%(E1). Tables 1, 2, 3 and 4 show the patients baseline characteristics, protocol related parameters and cycle outcomes, such as COH outcomes, Embryo transfer and pregnancy outcomes. There was significant difference regarding infertility years among groups A1, B1, C1, D1, E1 (P<0.05) with the longest in group E1. At the same time, no significant differences were detected in age, BMI, infertility factors, AMH level (Roche fully automatic electrochemical luminescence detector model e602; Roche Diagnostic GmbH, Germany) and antral follicle number (P > 0.05).

**Table 1:** Baseline Characteristics in LH level changes before administration of GnRH-ant

	A1	B1	C1	D1	E1	*P value*
No. of patients	395	179	99	19	29	
Age (years)	31.4 ± 4.9	31.3 ± 4.7	31.5 ± 5.0	30.8 ± 4.4	30.5 ± 4.9	0.902
Body mass index (kg/m ^2^)	21.4 ± 3.0	21.7 ± 3.1	21.8 ± 2.7	21.7 ± 2.3	22.3 ± 3.4	0.531
Duration of fertility (years)	3.4 ± 2.6	3.4 ± 2.6	3.7 ± 2.6	4.3 ± 1.6	3.8 ± 2.6	0.035
Etiology of infertility (%)						
Primary infertility	190/395	92/179	51/99	11/19	14/29	0.866
Secondary infertility	205/395	87/179	48/99	8/19	15/29	0.866
Female factor	273/395	141/179	77/99	15/19	23/29	0.058
Male factor	106/395	36/179	18/99	2/19	5/29	0.058
Combined	16/395	2/179	4/99	2/19	1/29	0.058
AMH	4.9 ± 3.9	5.4 ± 4.3	6.2 ± 4.7	5.0 ± 4.3	6.7 ± 4.4	0.052
Antral follicular count	13.0 ± 5.9	14.2 ± 6.7	14.3 ± 7.0	12.3 ± 4.7	15.3 ± 6.6	0.147

Note: Continuous variables are presented as means (± SD),Categorical variables are presented as percentage (their frequencies)

**Table 2:** Ovarian Stimulation Parameters in LH level changes before administration of GnRH-ant

	A1	B1	C1	D1	E1	*P value*
No. of patients	395	179	99	19	29	
Duration of stimulation (days)	8.7 ± 1.4	8.5 ± 1.5	8.6 ± 1.4	8.2 ± 1.3	8.8 ± 1.8	0.067
Total dose of rFSH (IU)	1669.0 ± 531.3	1627.5 ± 570.9	1580.1 ± 596.0	1663.2 ± 376.8	1643.1 ± 725.0	0.111
Hormone profile on the initiation day of rFSH						
FSH base (Iu/ml)	6.8 ± 1.7	6.7 ± 1.9	6.7 ± 2.3	6.1 ± 1.2	5.6 ± 2.0	0.001
LH base (Iu/ml)	6.3 ± 2.7	6.4 ± 3.1	6.2 ± 3.2	5.0 ± 3.0	5.8 ± 3.5	0.103
E2 base (Iu/ml)	35.9 ± 14.4	41.1 ± 19.2	42.6 ± 19.2	37.3 ± 13.8	36.3 ± 14.0	0.013
Duration of GnRH-ant (days)	5.4 ± 1.1	5.3 ± 1.2	5.1 ± 1.2	4.5 ± 1.1	5.2 ± 1.2	0.001
Total dose of GnRH-ant (mg)	1.3 ± 0.3	1.3 ± 0.3	1.3 ± 0.3	1.1 ± 0.3	1.4 ± 0.3	0.020
Hormone profile on the initiation day of GnRH-ant						
FSH (Iu/ml)	12.1 ± 3.9	12.3 ± 4.1	12.2 ± 3.8	15.5 ± 5.0	13.2 ± 5.8	0.036
LH (Iu/ml)	2.1 ± 1.1	3.9 ± 2.0	5.8 ± 3.2	6.9 ± 4.2	13.7 ± 12.1	<0.001
E2 (Iu/ml)	568.4 ± 387.2	758.3 ± 490.2	838.2 ± 528.5	984.6 ± 484.5	1008.2 ± 433.0	<0.001
Hormone profile on the hCG day						
FSH (Iu/ml)	13.4 ± 7.1	13.5 ± 10.4	12.0 ± 4.4	15.2 ± 5.5	12.1 ± 5.0	0.022
LH (Iu/ml)	3.6 ± 4.7	3.9 ± 2.5	4.7 ± 3.4	4.1 ± 2.9	6.1 ± 7.9	0.001
E2 (Iu/ml)	2717.0 ± 1421.7	3012.0 ± 1666.5	3260.8 ± 1767.2	3097.7 ± 1586.5	3792.7 ± 1652.5	0.002
P (Iu/ml)	0.9 ± 1.3	0.7 ± 0.4	0.8 ± 0.4	09 ± 0.4	0.8 ± 0.4	0.176
Endometrial thickness (mm) on the hCG day	10.9 ± 2.3	10.6 ± 2.4	10.5 ± 2.1	10.9 ± 2.1	10.9 ± 1.9	0.279

**Table 3:** COH outcomes in LH level changes before administration of GnRH-ant

	A1	B1	C1	D1	E1	*P value*
No. of patients	395	179	99	19	29	
Number of oocytes retrieved	12.2 ± 6.0	12.7 ± 7.3	13.0 ± 7.5	13.6 ± 7.8	15.6 ± 9.3	0.355
Follicular output rate (%)	78.9 ± 39.7	79.9 ± 80.7	77.1 ± 38.7	84.8 ± 33.1	77.7 ± 36.1	0.321
Number of Mature oocyte	9.3 ± 5.1	10.0 ± 6.5	10.6 ± 6.6	10.0 ± 6.6	11.8 ± 7.9	0.461
Number of 2PN fertilized oocytes	7.9 ± 4.6	8.3 ± 5.6	9.0 ± 5.9	8.6 ± 5.8	9.7 ± 6.1	0.498
2PN Fertilized rate (%)	73.0 ± 24.1	69.3 ± 24.5	73.9 ± 20.9	74.6 ± 26.4	68.1 ± 22.2	0.225
Number of cleavage	7.9 ± 4.6	8.3 ± 5.6	8.9 ± 5.9	8.6 ± 5.8	9.7 ± 6.1	0.494
Cleavage rate (%)	97.7 ± 14.3	96.5 ± 18.0	99.8 ± 1.7	94.2 ± 22.9	100 ± 0	0.257
Number of high quality embryos	4.3 ± 3.5	4.5 ± 4.2	4.7 ± 3.9	4.8 ± 4.6	4.9 ± 4.0	0.899
High-quality embryonic rate (%)	54.3 ± 33.0	52.7 ± 32.8	53.5 ± 31.2	52.2 ± 35.1	55.4 ± 32.3	0.981
No. of embryo available	5.2 ± 2.7	5.2 ± 2.7	5.7 ± 3.0	5.2 ± 3.0	5.7 ± 3.0	0.535

**Table 4:** Embryo transfer and pregnancy outcomes in LH level changes before administration of GnRH-ant

	A1	B1	C1	D1	E1	*P value*
No. of patients	395	179	99	19	29	
No. of ET patients	179	71	39	5	8	
Cancellation rate (%)	54.7	60.3	60.6	73.7	72.4	0.143
Implantation rate (%)	37.0	42.2	40.4	33.3	63.6	0.439
95%CI	0.310–0.429	0.324–0.519	0.272–0.535	-0.209-0.875	0.297–0.975	
D3 embryos Implantation rate (%)	27.0	36.2	30.8	40.0	57.1	0.287
95%CI	0.203–0.337	0.246–0.479	0.156–0.459	-0.280-1.080	0.077–1.066	
D5 embryos Implantation rate (%)	57.8	54.5	61.1	0	75.0	0.815
95%CI	0.470–0.687	0.366–0.725	0.362–0.861	-	-0.046-1.546	
Clinical pregnancy rate (%)	48.0	54.9	51.3	40.0	75.0	0.542
95%CI	0.407–0.554	0.431–0.668	0.349–0.677	-0.280-1.080	0.363–1.137	
Chemical pregnancy rate (%)	6.1	1.4	2.6	0	0	0.523
Early abortion rate (%)	10.5	15.4	10.0	0	0	0.850

Note: Categorical variables are presented as percentage (their frequencies)

The results showed that although duration of stimulation and the total dose of Gn in 5 groups had no statistical differences, the total dose of GnRH-ant and duration of antagonist in group D1 were lower than those in other groups (P < 0.05). There were significant differences in FSH and E2 levels among all groups during ovarian stimulation (P<0.05). while there were no statistical differences in progesterone (P) level and endometrial thickness in 5 groups on HCG trigger day (P>0.05).

It was found that there was no significant correlation between LH change before the antagonist initiation and COH outcome items among all groups (P > 0.05).

There were no statistically significant differences regarding the ET outcomes among all groups (P > 0.05).

### The effect of LH level changes after the initiation of GnRH-ant on the outcome of COH and ET

Tables 5, 6, 7 and 8 show a total of 721 first IVF/ICSI patient-cycles with 100 cycles (13.9%) in LH decreased more than 50% after the initiation of GnRH-ant (A2), 110 cycles (15.3%) in LH decreased 25-50% (B2), 178 cycles (24.7%) in LH change less than 25%(C2), 62 cycles (8.6%) in LH increased 25-50% (D2) and 271 cycles (37.6%) in LH increased more than 50%(E2). According to Kruskal-Wallis test, there were statistically significant differences in age, BMI, infertility type, AMH and AFC among all groups, with age and BMI higher in group E2 than those in other groups (P < 0.05) but AMH and AFC lower in group E2 (P < 0.001).

**Table 5:** Baseline Characteristics in LH level changes after initiation of GnRH-ant

	A2	B2	C2	D2	E2	*P value*
No. of patients	100	110	178	62	271	
Age (years)	30.3 ± 3.9	30.6 ± 4.6	31.0 ± 4.5	31.6 ± 5.1	32.2 ± 5.3	0.004
Body mass index (kg/m ^2^)	21.1 ± 3.0	20.9 ± 2.4	21.2 ± 2.4	21.6 ± 4.4	22.3 ± 2.9	<0.001
Duration of fertility (years)	3.6 ± 2.2	3.6 ± 2.4	3.7 ± 2.7	3.3 ± 2.5	3.4 ± 2.6	0.095
Etiology of infertility (%)						
Primary infertility	55/100	51/110	103/178	29/62	120/271	0.044
Secondary infertility	45/100	59/110	75/178	33/62	151/171	0.044
Female factor	76/100	87/110	126/178	45/62	195/271	0.592
Male factor	21/100	22/110	44/178	13/62	67/271	0.592
Combined	3/100	1/110	8/178	4/62	9/271	0.592
AMH	7.5 ± 4.1	6.4 ± 5.0	5.7 ± 4.1	4.8 ± 3.8	3.9 ± 3.4	<0.001
Antral follicular count	16.6 ± 5.4	15.0 ± 6.8	13.8 ± 6.3	11.9 ± 6.0	12.0 ± 5.9	<0.001

Note: Continuous variables are presented as means (± SD),Categorical variables are presented as percentage (their frequencies)

**Table 6:** Ovarian Stimulation Parameters in LH level changes after initiation of GnRH-ant

	A2	B2	C2	D2	E2	*P value*
No. of patients	100	110	178	62	271	
Duration of stimulation (days)	8.4 ± 1.4	8.6 ± 1.3	8.5 ± 1.4	8.3 ± 0.14	8.9 ± 1.5	0.004
Total dose of rFSH (IU)	1379.0 ± 445.1	1549.8 ± 540.4	1556.1 ± 439.3	1590.3 ± 558.7	1853.4 ± 596.1	<0.001
Hormone profile on the initiation day of rFSH						
FSH base (Iu/ml)	6.1 ± 1.7	6.4 ± 1.7	7.0 ± 2.0	7.0 ± 1.7	6.8 ± 1.9	<0.001
LH base (Iu/ml)	7.6 ± 3.8	6.8 ± 3.0	6.6 ± 2.8	6.0 ± 2.1	5.3 ± 2.4	<0.001
E2 base (Iu/ml)	39.8 ± 18.2	40.0 ± 17.2	41.1 ± 17.9	35.5 ± 17.3	35.5 ± 14.0	0.002
Duration of GnRH-ant (days)	5.2 ± 1.1	5.2 ± 1.2	5.3 ± 1.2	5.2 ± 1.2	5.4 ± 1.2	0.457
Total dose of GnRH-ant (mg)	1.3 ± 0.3	1.3 ± 0.3	1.3 ± 0.3	1.3 ± 0.3	1.3 ± 0.3	0.570
Hormone profile on the initiation day of GnRH-ant						
FSH (Iu/ml)	12.1 ± 3.4	12.6 ± 4.2	12.5 ± 4.1	12.3 ± 4.2	12.2 ± 4.2	0.885
LH (Iu/ml)	7.9 ± 7.9	4.6 ± 2.8	3.2 ± 2.2	2.6 ± 1.4	2.2 ± 1.4	<0.001
E2 (Iu/ml)	1090.8 ± 440.9	862.2 ± 428.7	713.8 ± 464.6	586.8 ± 394.4	457.0 ± 335.3	<0.001
Hormone profile on the hCG day						
FSH (Iu/ml)	11.1 ± 3.7	12.6 ± 4.6	14.2 ± 13.3	13.5 ± 4.9	13.5 ± 4.4	<0.001
LH (Iu/ml)	2.3 ± 1.7	2.8 ± 1.8	3.2 ± 2.1	3.6 ± 1.9	5.5 ± 6.1	<0.001
E2 (Iu/ml)	4016.8 ± 1644.7	3283.1 ± 1478.1	2903.1 ± 1524.9	2438.3 ± 1350.6	2484.4 ± 1415.6	<0.001
P (Iu/ml)	0.8 ± 0.5	0.9 ± 1.2	0.8 ± 0.4	0.6 ± 0.3	0.8 ± 1.3	0.049
Endometrial thickness (mm) on the hCG day	10.4 ± 2.3	10.8 ± 2.5	10.8 ± 2.0	10.0 ± 2.1	10.9 ± 2.3	0.486

**Table 7:** COH outcomes in LH level changes after initiation of GnRH-ant

	A2	B2	C2	D2	E2	*P value*
No. of patients	100	110	178	62	271	
Number of oocytes retrieved	16.4 ± 7.1	14.5 ± 7.1	11.9 ± 6.0	11.8 ± 7.5	11.0 ± 6.1	<0.001
Follicular output rate (%)	86.2 ± 40.5	83.7 ± 97.1	77.1 ± 36.2	81.2 ± 67.9	75.2 ± 31.5	0.208
Number of Mature oocyte	12.8 ± 6.9	11.7 ± 6.3	9.2 ± 5.3	9.1 ± 5.7	8.4 ± 5.0	<0.001
Number of 2PN fertilized oocytes	10.6 ± 5.8	10.1 ± 5.5	7.9 ± 4.8	7.7 ± 4.8	7.0 ± 4.6	<0.001
2PN Fertilized rate (%)	69.7 ± 23.0	75.2 ± 20.1	72.1 ± 23.2	72.8 ± 22.9	71.5 ± 25.9	0.631
Number of cleavage	10.6 ± 5.8	10.0 ± 5.5	7.9 ± 4.8	7.6 ± 4.8	7.0 ± 4.6	<0.001
Cleavage rate (%)	97.9 ± 14.1	97.9 ± 13.5	98.0 ± 13.0	96.7 ± 17.8	97.5 ± 15.0	0.924
Number of high quality embryos	5.6 ± 3.8	5.8 ± 4.5	4.6 ± 3.4	4.3 ± 3.9	3.5 ± 3.4	<0.001
High-quality embryonic rate (%)	55.4 ± 18.9	55.2 ± 33.2	59.1 ± 33.0	54.0 ± 29.0	49.0 ± 33.8	0.047
No. of embryo available	6.4 ± 2.8	5.9 ± 2.7	5.4 ± 2.8	4.6 ± 2.5	4.7 ± 2.7	<0.001

**Table 8:** Embryo transfer and pregnancy outcomes in LH levels after initiation of GnRH-ant

	A2	B2	C2	D2	E2	*P value*
No. of patients	100	110	178	62	271	
No. of ET patients	23	26	83	29	142	
Cancellation rate (%)	77.0	76.4	53.4	53.2	47.6	<0.001
Implantation rate (%)	48.1	35.3	41.5	39.0	37.5	0.803
95%CI	0.280–0.683	0.184–0.522	0.326–0.503	0.234–0.546	0.309–0.441	
D3 Implantation rate (%)	44.4	28.6	33.7	32.1	27.9	0.745
95%CI	0.039–0.850	0.075–0.496	0.237–0.437	0.137–0.506	0.206–0.350	
D5 Implantation rate (%)	50.0	46.2	61.8	53.8	60.7	0.803
95%CI	0.244–0.756	0.148–0.775	0.446–0.790	0.225–0.852	0.480–0.733	
Clinical pregnancy rate (%)	43.5	42.3	50.6	48.3	52.8	0.832
95%CI	0.216–0.654	0.220–0.627	0.396–0.616	0.289–0.676	0.445–0.611	
Chemical pregnancy rate (%)	8.7	3.8	1.2	3.4	4.9	0.335
Early abortion rate (%)	10.0	0	7.1	7.1	16	0.512

Note: Categorical variables are presented as percentage (their frequencies)

The days of ovarian stimulation and the total amount of Gn in group A2 were lower than those in other groups (P = 0.004, P < 0.001). And FSH level in group A2 was the lowest on the initiation day of stimulation and trigger day (P < 0.001).

Among the 5 groups, the number of oocytes retrieved, mature oocytes, 2PN fertilized oocytes, embryo cleavage and the numbers of embryo available in group A2 were significantly higher than those in other groups (P < 0.05). The group A2 had the highest cancellation rate (77.0%) (P < 0.001), while there were no significant differences in ET outcomes among all groups (P > 0.05). According to multiple linear regression analysis, the number of oocytes retrieved were significantly affected by patients’ age, AMH and AFC. While the LH level changes after the addition of antagonists had no significant impact on the number of oocytes retrieved. However, patients’ age, AMH, AFC and the LH level changes after the addition of antagonists had no significant impact on the number of available embryos (Supplementary Tables 1–2). According to binary logistic analysis, LH level changes after the initiation of antagonist did not have a significant effect on clinical pregnancy, but age had a significant effect on clinical pregnancy (Supplementary Table 3).

## Discussion

It has been demonstrated that < 1% of LH receptors being occupied is enough to elicit a normal steroidogenic response [10]. However, there is no consensus on the optimal clinical LH threshold range for COH in assisted reproductive technology (ART) [11]. In the study by Bosch E and coworkers [12], no differences were observed between the number of oocyte retrived or the fertilization, implantation, and pregnancy rates of different LH concentrations on days 3, 6, and 8 of stimulation and on the day of hCG, what was also supported by other similar studies [13].

During the ovarian stimulation process of GnRH-ant protocol, 55%(395/721) of the patients showed a spontaneously significant decrease in LH level before the addition of the antagonist, and only a few patients (37/721) had a significant increase in LH level. The significant decrease in LH level before the addition of antagonists may be related to the production of gonadotrophin surge-attenuating factor (GnSAF). Previous studies [14, 15] have found that in the process of assisted reproduction using exogenous gonadotropin to promote follicle development, exogenous gonadotropin was found to stimulate the ovarian production of an uncharacterized hormone known by its specific effect of reducing pituitary responsiveness to GnRH. This hormone has been called gonadotrophin surge-attenuating factor (GnSAF). It regulates LH secretion by reducing the sensitivity of the pituitary to GnRH and antagonizing the stimulatory effects of oestradiol on GnRH-induced LH secretion. The main role of GnSAF is probably the negative regulation of pulsatile LH secretion, mainly during the first half of the follicular phase. What’s more, the results of this study showed that the change of LH level before the addition of the antagonist did not affect the outcome of the COH and pregnancy outcome. It was basically consistent with the study of Vanetik et al. [16], they proposed that there was no statistical difference in pregnancy outcome when LH level increased or decreased on the 5th day compared with that on the initiation day during COH. While the results of a retrospective study involving 2116 fresh ET cycles showed that before antagonist addition, the oocytes retrieved rate and fertilization rate in the group with LH increased were lower than those in the group with LH decreased [17], but there was no statistical difference in pregnancy-related outcomes among the groups.

LH level changes after the addition of antagonists had a significant difference in the outcome of ovarian stimulation with the number of oocyte retrieved and available embryos in group A2 significantly higher than those in other groups. But, no significant difference was found between LH level changes and embryo transfer result. It was revealed in our study the total rFSH dose was significantly lower in people with LH decreased ≥ 50% after the addition of antagonists during COH, while their E2 level was relatively higher on trigger day. We consider that it was associated with a better ovarian response in people with significantly decreased LH level after antagonist addition, for AMH and AFC were higher in them, also the age was younger in them. Existing studies have demonstrated that ovarian responsiveness is generally assessed by ovarian markers such as antral follicle count (AFC) and anti-Müllerian (AMH), in conjunction with age, in order to predict poor, normal or hyper-response [18]. It has been proved that with the increase of estrogen level (E2 ≤ 4 800 pg/mL) in the GnRH-ant protocol, both the number of oocyte retrieved and embryos obtained increased [19]. Similarly, we found people who with significantly decreased LH had a higher average level of E2, therefore had a relatively better COH outcomes. Besides, the number of oocyte retrieved, mature oocytes, 2PN fertilized oocytes, embryos cleavage and the numbers of embryo available were negatively correlated with LH level changes. In the study of Scheffer and coworkers [20], they found that age was negatively correlated with the quality of D3 and D5 embryos, but AMH and AFC were positively correlated with the quality of D3 and D5 embryos. Here in this study we also found that the number of high-quality embryos and high-quality embryonic rate were lower in patients with significantly increased LH level, which was related to that patient with advanced age, low AMH and low AFC was more prone to appear LH significantly increase.

There are many factors affecting the outcomes of COH and pregnancy. Such as E2, LH and P levels on trigger day as well as age and BMI all have certain influence on the outcomes of ovulation stimulation and pregnancy, while LH level on trigger day is negatively correlated with the number of oocytes retrieved, but has no significant correlation with the outcome of pregnancy [21].The conclusion is consistent with Ji Hui and coworkers’ results [22], they proposed that low LH levels on trigger day can predict higher the number of oocyte retrieved, but no effect on early miscarriage rate or clinical pregnancy rate. Similarly, we observed that there were no significant differences in pregnancy outcomes among different LH change groups after GnRH-ant addition (P > 0.05). However, Younis JS and coworkers found that a decrease of LH level > 50% significantly reduced the embryo transfer outcome [23]. And in the study by Geng Y and coworkers [24], the clinical pregnancy rates were reduced in high ovarian response patients with LH surge during ovulation stimulation in GnRH-ant protocol.

## Conclusion

In conclusion, this study demonstrates that LH level changes before GnRH-ant initiation had no effect on COH and ET outcomes. The number of oocyte retrieved, mature oocytes, fertilized oocytes, embryos cleavage, high quality embryos, high-quality embryonic rate and the numbers of embryo available have significant differences with the change of LH after GnRH-ant initiation, but without influence on the pregnancy outcome. Therefore, the LH level changes in GnRH antagonist protocol can predict the outcomes of ovulation stimulation to a certain extent, but it cannot be used to predict the outcome of clinical pregnancy. What’s more, the limitation of this study lies in the fact that it is a retrospective study, which is influenced by unnoticed bias or confounding factors. Meanwhile, on account of the unequal sample size among the groups, the sample may not accurately reflect the overall situation. Hence, more comparative studies are needed in the future.

### Electronic supplementary material

Below is the link to the electronic supplementary material.

Supplementary Material 1

## Data Availability

The relevant data needed for the article is presented in the article content and in the attached file table.

## Abbreviations

ART: assisted reproductive technology
COH: controlled ovarian hyperstimulation
E2: Estrogen
ET: embryo transfer
FSH: Follicle stimulating hormone
GnRH-ant: gonadotropin-releasing hormone antagonist
Gn: gonadotropin
GnSAF: gonadotrophin surge-attenuating factor
hCG: human chorionic gonadotropin
ICSI: intracytoplasmic sperm injection
IVF: in vitro fertilization
LH: Luteinizing hormone
OHSS: ovarian hyperstimulation syndrome
PN: pronucleus
P: Progesterone
rhCG: recombinant human chorionic gonadotropin
SD: standard deviation
