# Supplementary material for: The effect of luteinizing hormone changes in GnRH antagonist protocol on the outcome of controlled ovarian hyperstimulation and embryo transfer
Source: BMC Pregnancy Childbirth. 2023 Aug 23;23:604. doi: 10.1186/s12884-023-05916-8 (PMC10464317; doi:10.1186/s12884-023-05916-8)
Supplement: Supplementary file 1 — Supplementary Material 1 [file 12884_2023_5916_MOESM1_ESM.docx]

| Supplementary table 1. Multiple linear regression analysis results of predictions on the number of oocyte retrieved | | | | | | |
| --- | --- | --- | --- | --- | --- | --- |
|  | *Unstandardized B* | *Standardized*  *Coefficients Beta* | *t* | *P value* | *F* | *Adjusted R^2^* |
| Age | -0.158 | -0.110 | -3.112 | 0.002 | 58.443 | 0.324 |
| BMI | -0.098 | -0.043 | -1.358 | 0.175 |  |  |
| AMH | 0.342 | 0.210 | 5.055 | 0.000 |  |  |
| AFC | 0.393 | 0.365 | 8.520 | 0.000 |  |  |
| LH level changes  after initiation of GnRH-ant | 0.088 | 0.097 | 0.028 | 0.361 |  |  |

| Supplementary table 2. Multiple linear regression analysis results of predictions on the number of available embryos | | | | | | |
| --- | --- | --- | --- | --- | --- | --- |
|  | *Unstandardized B* | *Standardized*  *Coefficients Beta* | *t* | *P value* | *F* | *Adjusted R^2^* |
| Age | 0.001 | 0.006 | 0.129 | 0.897 | 0.372 | -0.005 |
| BMI | 0.002 | 0.007 | 0.188 | 0.851 |  |  |
| AMH | 2.166E-5 | 0.000 | 0.002 | 0.998 |  |  |
| AFC | -0.002 | -0.014 | -0.273 | 0.785 |  |  |
| LH level changes  after initiation of GnRH-ant | 0.010 | 0.026 | 0.687 | 0.492 |  |  |

| Supplementary table 3. Binary logistic regression analysis results of clinical pregnancy outcome | | | | | | |
| --- | --- | --- | --- | --- | --- | --- |
|  | *B* | *S.E.* | *Wald* | *df* | *Sig.* | *Exp(B)* |
| LH level changes  after initiation of GnRH-ant |  |  | 4.427 | 4 | .351 |  |
| LH level changes  after initiation of GnRH-ant(1) | .951 | .567 | 2.815 | 1 | .093 | 2.587 |
| LH level changes  after initiation of GnRH-ant(2) | .830 | .494 | 2.829 | 1 | .093 | 2.294 |
| LH level changes  after initiation of GnRH-ant(3) | .149 | .299 | .248 | 1 | .618 | 1.161 |
| LH level changes  after initiation of GnRH-ant(4) | .162 | .418 | .151 | 1 | .697 | 1.176 |
| LH level changes  before initiation of GnRH-ant |  |  | 6.122 | 4 | .190 |  |
| LH level changes  before initiation of GnRH-ant(1) | 2.080 | .936 | 4.937 | 1 | .026 | 8.001 |
| LH level changes  before initiation of GnRH-ant(2) | 1.649 | .926 | 3.174 | 1 | .075 | 5.203 |
| LH level changes  before initiation of GnRH-ant(3) | 1.711 | .945 | 3.281 | 1 | .070 | 5.536 |
| LH level changes  before initiation of GnRH-ant(4) | 1.917 | 1.297 | 2.185 | 1 | .139 | 6.803 |
| Age | .072 | .028 | 6.609 | 1 | .010 | 1.074 |
| BMI | -.031 | .039 | .641 | 1 | .423 | .969 |
| AMH | .041 | .050 | .695 | 1 | .404 | 1.042 |
| AFC | -.018 | .029 | .404 | 1 | .525 | .982 |
| Constant | -3.645 | 1.627 | 5.021 | 1 | .025 | .026 |
